# Supplementary material for: Comprehensive functional core microbiome comparison in genetically obese and lean hosts under the same environment
Source: Commun Biol. 2021 Nov 1;4:1246. doi: 10.1038/s42003-021-02784-w (PMC8560826; doi:10.1038/s42003-021-02784-w)
Supplement: Supplementary file 2 — Supplementary Material [file 42003_2021_2784_MOESM2_ESM.pdf]

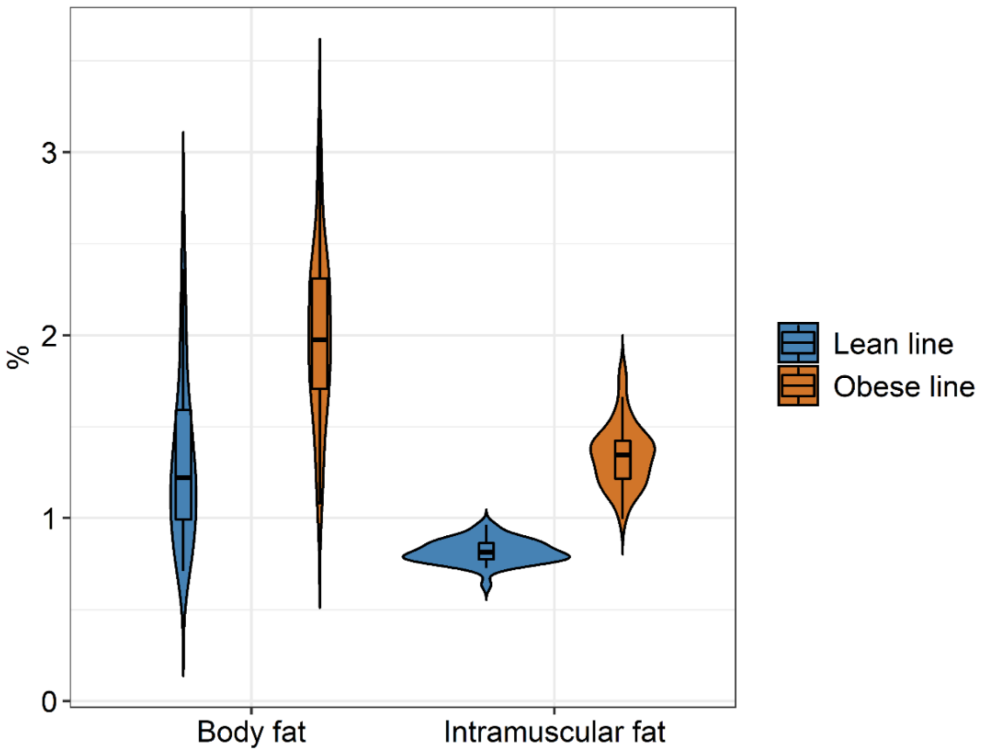

Supplementary Figure 1. Fat content of obese and lean genetic lines. Body fat percentage and intramuscular fat content in muscle *Longissimus thoracis et lumborum*. Body fat percentage (%) estimated as (perirenal + scapular fat weight (g) / reference carcass weight (g)) x 100 and intramuscular fat content (%) measured as g lipids /100 g *Longissimus thoracis et lumborum* muscle. The obese line presented 0.53 (P_0^1=1.00) g of lipids/100g of *LTL* muscle more than the lean line, equivalent to 4.14 phenotypic standard deviations (SD) of the trait, and the distributions of both lines scarcely overlap. A correlated response to selection in the main fat depots in rabbits confirms the common genetic basis regulating lipid deposition in intramuscular and main body fat reservoirs, independently on whether body fat is expressed as weight (the obese line presenting +5.11(P_0^1=1.00) and +0.60 g (P_0^1=0.95) of perirenal and scapular fat weights respectively) or as a percentage of total carcass weight (+0.69%, P_0^1=1.00). [1]$P_{0,}$ probability of the differences between lines in absolute value being higher than zero.

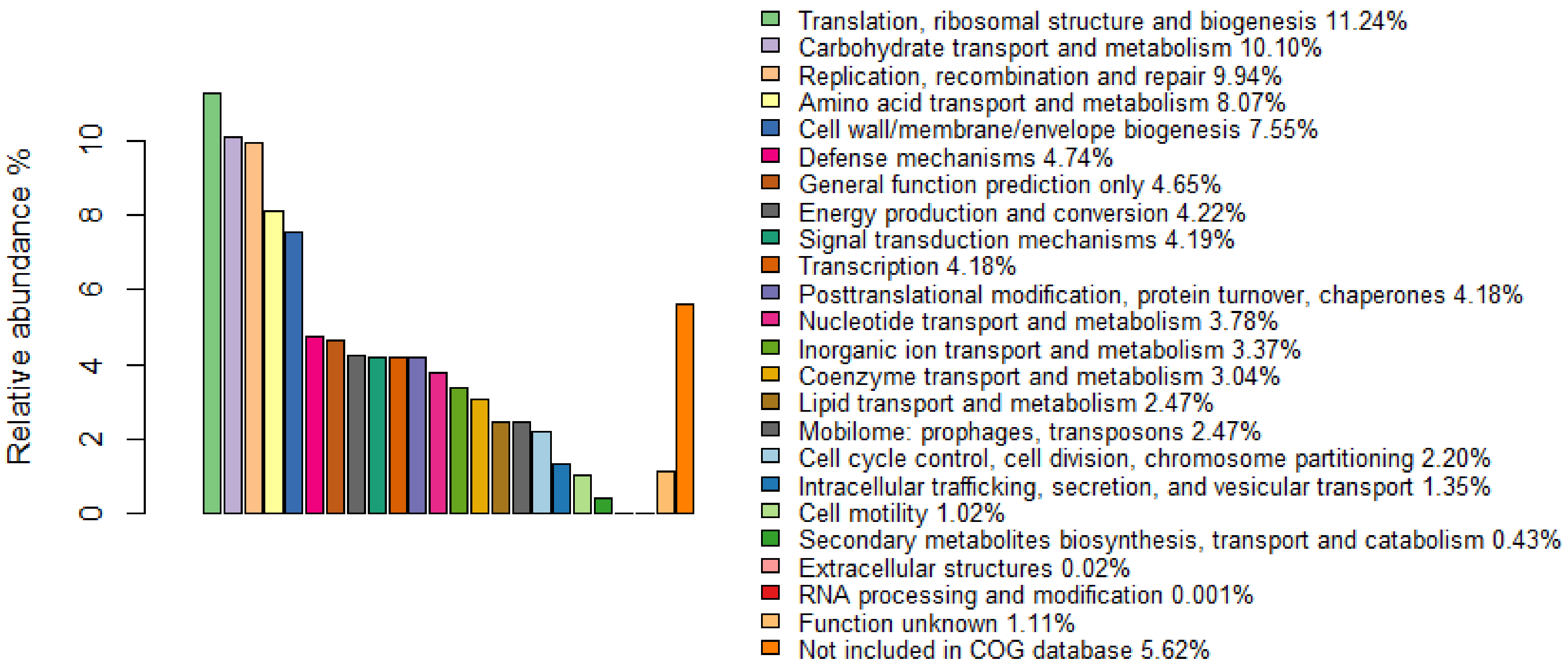

Supplementary Figure 2. Functional composition of rabbit caecal microbiome. Functional composition of rabbit caecal microbiome obtained by summing relative abundances of microbial genes classified within the same Cluster of Orthologous Groups of proteins (COG). Metabolism and transport of carbohydrates (352 MGs), amino acids (313 MGs), and cell wall/membrane, envelope biogenesis (315 MGs) COGs accumulate the largest number of microbial genes and represented 10.1%, 8.1%, and 7.6% cumulative relative abundances, respectively. Translation, ribosomal structure and biogenesis and replication, combination and repair comprise a considerable proportion of the total abundance (11.2% and 9.9%, respectively), although the total number of MGs classified within these COGs is smaller (243 and 137 MGs, respectively).

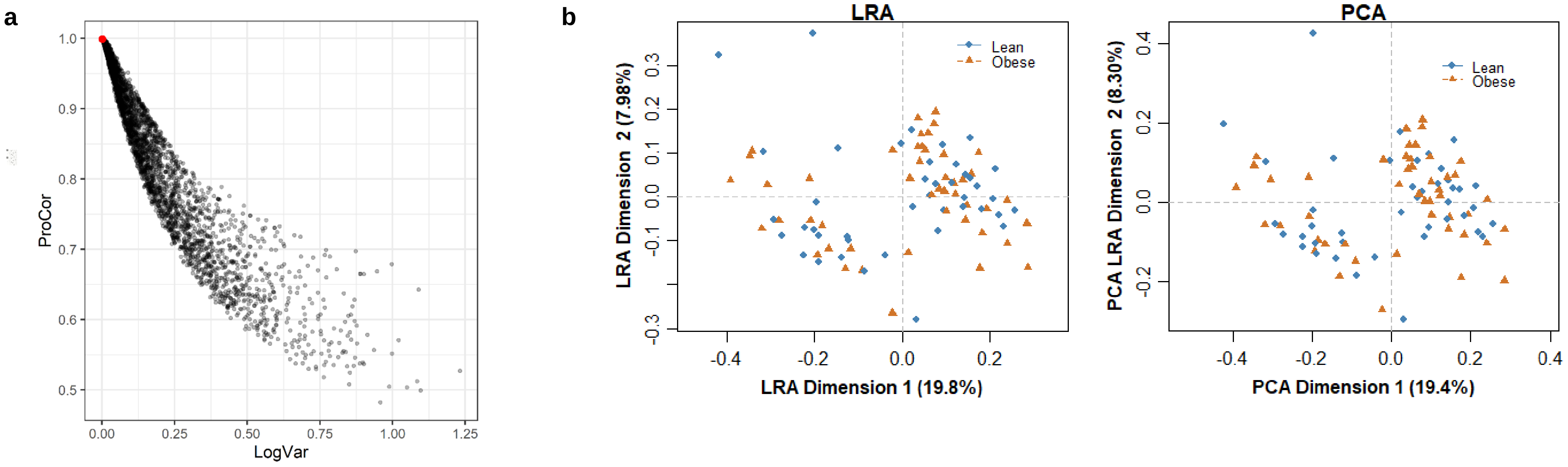

Supplementary Figure 3. Selection of the reference microbial gene for the additive log ratio transformation. a. Selection of the reference housekeeping microbial gene 'small subunit ribosomal protein S1' (*RP-S1*) -highlighted in red- as a denominator for additive log-ratio transformation based on minimal variance of log-transformed relative abundance (LogVar) and maximal Procrustes correlation with the whole pairwise log-ratio geometry (ProCor). **b.** Sample plot showing the similarity between a log-ratio analysis including all possible pairwise log-ratios (LRA, left) and principal components analysis of the set of additive log-ratios using *RP-S1* as denominator (PCA, right).

Supplementary Table 1. Descriptive statistics and differences between obese and lean lines for lipidic traits in the 10th generation of selection.

	Median	CV	SD	Obese line[3]	$HPD_{95\%}$[4]	Lean line[3]	$HPD_{95\%}$[4]	Obese - Lean line[3]	$HPD_{95\%}$[4]	P_0[5]
Intramuscular fat, g fat/100g muscle[1]	1.04	12.42	0.13	1.30	[1.25, 1.35]	0.77	[0.72, 0.82]	0.53	[0.48, 0.59]	1
Perirenal fat weight, g	9.81	38.11	3.74	12.36	[10.9, 13.8]	7.26	[5.64, 8.76]	5.11	[3.52 ,6.70]	1
Scapular fat weight, g	4.06	39.21	1.59	4.36	[3.75, 4.97]	3.76	[3.10, 4.41]	0.60	[-0.11, 1.26]	0.95
Body fat, %[2]	1.65	28.20	0.46	1.99	[1.82, 2.16]	1.30	[1.39, 1.75]	0.69	[0.49, 0.88]	1

[1]Intramuscular fat measured in *Longissimus thoracis et lumborum* muscle

[2]Body fat (%) estimated as perirenal + scapular fat weight (g) / reference carcass weight (g)

[3]Median of the marginal posterior distribution of the differences between lines.

[4]Higher posterior density interval enclosing 95% probabilty of the marginal posterior distribution of the differences between lines.

[5]Probability of the marginal posterior distribution of the differences between lines of being positive.
