# Supplementary material for: Comprehensive functional core microbiome comparison in genetically obese and lean hosts under the same environment
Source: Commun Biol. 2021 Nov 1;4:1246. doi: 10.1038/s42003-021-02784-w (PMC8560826; doi:10.1038/s42003-021-02784-w)
Supplement: Supplementary file 3 — Description of Additional Supplementary Files [file 42003_2021_2784_MOESM3_ESM.pdf]

Description of Additional Supplementary Files

File name: Supplementary Data 1
Description: Microbial gene composition in rabbit cecum.

File name: Supplementary Data 2
Description: Parameters from the Partial Least Squares Discriminant analysis model between obese and lean lines by using 240 microbial gene additive-log ratio transformed abundances.

File name: Supplementary Data 3
Description: Parameters from the Partial Least Squares analysis model explaining intramuscular fat content (mg/ 100g muscle) by using 230 microbial gene additive-log ratio transformed abundances.

File name: Supplementary Data 4
Description: Differences between lines in the abundance of the 122 microbial gene additive-log ratio transformed abundances overlapping in Partial Least Squares Discriminant analysis and Partial Least Squares models.

File name: Supplementary Data 5
Description: Parameters from the Partial Least Squares Discriminant analysis model between obese and lean lines by using 279 microbial gene relative abundances.

File name: Supplementary Data 6
Description: Parameters from the Partial Least Squares analysis model explaining intramuscular fat content (mg/ 100g muscle) by using 344 microbial gene relative abundances.

File name: Supplementary Data 7
Description: Microbial gene abundances overlapping in Partial Least Squares Discriminant analysis and Partial Least Squares models when using relative abundance transformation.

File name: Supplementary Data 8
Description: Top ten microbial taxa harbouring the greatest number of unique proteins clustered in the Kegg Orthologous groups (KO) or microbial genes explaining intramuscular fat content.

File name: Supplementary Data 9
Description: Parameters from the Partial Least Squares analysis model explaining body fat percentage by using 184 microbial gene additive-log ratio transformed abundances.
