# Supplementary material for: Comprehensive functional core microbiome comparison in genetically obese and lean hosts under the same environment
Source: Commun Biol. 2021 Nov 1;4:1246. doi: 10.1038/s42003-021-02784-w (PMC8560826; doi:10.1038/s42003-021-02784-w)
Supplement: Supplementary file 13 — Reporting Summary [file 42003_2021_2784_MOESM13_ESM.pdf]

nature portfolio

Corresponding author(s): AGUSTIN BLASCO

Last updated by author(s): Jun 3, 2021

Reporting Summary

Nature Portfolio wishes to improve the reproducibility of the work that we publish. This form provides structure for consistency and transparency in reporting. For further information on Nature Portfolio policies, see our Editorial Policies and the Editorial Policy Checklist.

Statistics

For all statistical analyses, confirm that the following items are present in the figure legend, table legend, main text, or Methods section.

n/a	Confirmed	
☐	☒	The exact sample size (*n*) for each experimental group/condition, given as a discrete number and unit of measurement
☐	☒	A statement on whether measurements were taken from distinct samples or whether the same sample was measured repeatedly
☒	☐	The statistical test(s) used AND whether they are one- or two-sided<br>*Only common tests should be described solely by name; describe more complex techniques in the Methods section.*
☒	☐	A description of all covariates tested
☐	☒	A description of any assumptions or corrections, such as tests of normality and adjustment for multiple comparisons
☐	☒	A full description of the statistical parameters including central tendency (e.g. means) or other basic estimates (e.g. regression coefficient) AND variation (e.g. standard deviation) or associated estimates of uncertainty (e.g. confidence intervals)
☒	☐	For null hypothesis testing, the test statistic (e.g. *F*, *t*, *r*) with confidence intervals, effect sizes, degrees of freedom and *P* value noted<br>*Give P values as exact values whenever suitable.*
☐	☒	For Bayesian analysis, information on the choice of priors and Markov chain Monte Carlo settings
☒	☐	For hierarchical and complex designs, identification of the appropriate level for tests and full reporting of outcomes
☒	☐	Estimates of effect sizes (e.g. Cohen's *d*, Pearson's *r*), indicating how they were calculated

Our web collection on statistics for biologists contains articles on many of the points above.

Software and code

Policy information about availability of computer code

Data collection	No software was used for collecting data
Data analysis	SqueezeMeta, SIMCA 15, KEGGREST, Bowtie2

For manuscripts utilizing custom algorithms or software that are central to the research but not yet described in published literature, software must be made available to editors and reviewers. We strongly encourage code deposition in a community repository (e.g. GitHub). See the Nature Portfolio guidelines for submitting code & software for further information.

Data

Policy information about availability of data

All manuscripts must include a data availability statement. This statement should provide the following information, where applicable:

- Accession codes, unique identifiers, or web links for publicly available datasets
- A description of any restrictions on data availability
- For clinical datasets or third party data, please ensure that the statement adheres to our policy

All data are available on request

Field-specific reporting

Life sciences study design

All studies must disclose on these points even when the disclosure is negative.

Sample size	Number of males (10) and females (60) per line was determined in order to provide enough samples for the experiment (in practice, 50 per line, half of each sex), avoiding inbreeding (less than 1.5% per generation). Finally we obtained 45 samples per line that were contemporary.
Data exclusions	One animal from the lean line was removed from the PLS and PLS-DA analyses for being detected as an outlier based on the distance of each individual to the model (DMOD criterion)
Replication	WE USED CROSS-VALIDATION IN ALL PLS AND PLS-DA ANALYSES
Randomization	SAMPLES COME FROM TWO DIVERGENT LINES SELECTED BY INTRAMUSCULAR FAT. WITHIN LINE, SAMPLES FROM TWO OFFSPRING (MALE AND FEMALE) CHOSEN AT RANDOM FROM EACH DAM WERE USED
Blinding	ONLY TWO GROUPS WERE USED, DIVERGENTLY SELECTED LINES FOR INTRAMUSCULAR FAT, BLINDING WAS NOT REQUIRED FOR ALLOCATION OF SAMPLES

Reporting for specific materials, systems and methods

We require information from authors about some types of materials, experimental systems and methods used in many studies. Here, indicate whether each material, system or method listed is relevant to your study. If you are not sure if a list item applies to your research, read the appropriate section before selecting a response.

Materials & experimental systems

n/a	Involved in the study
☒	☐ Antibodies
☒	☐ Eukaryotic cell lines
☒	☐ Palaeontology and archaeology
☐	☒ Animals and other organisms
☒	☐ Human research participants
☒	☐ Clinical data
☒	☐ Dual use research of concern

Methods

n/a	Involved in the study
☒	☐ ChIP-seq
☒	☐ Flow cytometry
☒	☐ MRI-based neuroimaging

Animals and other organisms

Policy information about studies involving animals; ARRIVE guidelines recommended for reporting animal research

Laboratory animals	RABBIT. SYNTHETIC STRAINS DIVERGENTLY SELECTED FOR INTRAMUSCULAR FAT
Wild animals	*Provide details on animals observed in or captured in the field; report species, sex and age where possible. Describe how animals were caught and transported and what happened to captive animals after the study (if killed, explain why and describe method; if released, say where and when) OR state that the study did not involve wild animals.*
Field-collected samples	NO SAMPLES COLLECTED FROM THE FIELD
Ethics oversight	All experimental procedures involving animals were approved by the Universitat Politècnica de València Research Ethics Committee, according to council directive 2010/63/EU (European Commission Directive, 2010).

Note that full information on the approval of the study protocol must also be provided in the manuscript.
